# Root condensed tannins vary over time, but are unrelated to leaf tannins

**DOI:** 10.1093/aobpla/ply044

**Published:** 2018-07-23

**Authors:** Margarete A Dettlaff, Valerie Marshall, Nadir Erbilgin, James F Cahill

**Affiliations:** 1 Department of Biological Sciences, University of Alberta, Edmonton, Alberta, Canada; 2 Department of Renewable Resources, University of Alberta, Edmonton, Alberta, Canada

**Keywords:** Above-ground plant defence, aspen, below-ground plant defence, seasonality

## Abstract

Although the negative effects of root herbivores on plant fitness are expected to be similar to those of above-ground herbivores, the study of below-ground plant defences is limited compared to the rich literature on above-ground defences. Current theory predicts that concentrations of defensive chemicals above- and below-ground should be correlated, as the evolutionary drivers that shape plant defence are similar across the whole plant. We conducted a field study to measure root condensed tannin concentrations in *Populus tremuloides*, and determine how they related to leaf condensed tannin concentrations, tree position within the stand (edge vs. interior), tree size, and time of year. Overall, root tannin concentrations were substantially lower than leaf tannin concentrations. At individual sampling periods, root and leaf tannin concentrations were uncorrelated with each other, and did not vary with stand position or size. Across the growing season both root and leaf tannin concentrations did show similar trends, with both highest in the early summer, and declining through mid-summer and fall. These results suggest that the mechanisms that influence leaf and root tannin levels in aspen are independent within individual stems, possibly due to different evolutionary pressures experienced by the different tissue types or in response to localized (roots vs. foliage) stressors. However, across individual stems, the similar patterns in chemical defence over time, independent of plant size or stand position indicate that larger scale processes can have consistent effects across individuals within a population, such as the relative investment in defence of tissues in the spring versus the fall. Overall, we conclude that using theories based on above-ground defence to predict below-ground defences may not be possible without further studies examining below-ground defence.

## Teaser

Although the negative effects of root herbivores on plant fitness are expected to be similar to those of above-ground herbivores, the study of below-ground plant defences is limited compared to the rich literature on above-ground defences. Given that the effects of above- and below-ground herbivores are expected to be similar, we would expect plants to defend their above- and below-ground tissues to a similar degree. To address this question, this study investigates the relationship between leaf and root tannins in *Populus tremuloides* across three time points.

## Introduction

The question of how plants defend above-ground tissues has received substantial research attention (Agrawal and Fishbein 2006; Chen 2008; Mithöfer and Boland 2012; Fürstenberg-Hägg *et al.* 2013), grounded in a well-developed body of theory (Loomis 1932; McKey 1979; Rhoades 1979; Bryant *et al.* 1983). However, our understanding of how plants defend themselves below-ground is limited to only a few studies (Moore and Johnson 2017) and additional studies are needed (Barbehenn and Peter Constabel 2011). We have only begun to apply theories of above-ground plant chemical defence to below-ground tissues (Kaplan *et al.* 2008; Rasmann and Agrawal 2008), and the extent to which below-ground defence may require new or modified theory is poorly understood, particularly in the context of below-ground chemical defences (Rasmann and Agrawal 2008). This is a striking gap, as the few studies that have compared the effects of above- and below-ground herbivory on plants indicate that they can have equivalent fitness consequences for the entire plant (Blossey and Hunt-Joshi 2003; Wurst *et al.* 2008), and are similarly vulnerable to stress (Kaplan *et al.* 2008).

Key to developing a theory to understand whole-plant chemical defence strategies will be more empirical data characterizing root and shoot defensive traits within individual plants. A first prediction would suggest that since plants in theory experience similar evolutionary pressures above- and below-ground, chemical defence allocation would then be a similar across the whole plant (Kaplan *et al.* 2008; Parker *et al.* 2012). However, evidence from non-defence-related root and leaf traits suggests that there is no general pattern in plants; some are similar across the whole plant, and some are tissue specific (Craine *et al.* 2001, 2005; Ryser 2006; Kembel and Cahill 2011; Fujii *et al.* 2016). Complicating the issue is that many chemical defences within species are highly variable due to prior encounters with natural enemies (Chen 2008), plant age (Bowers and Stamp 1993; Donaldson *et al.* 2006b), local conditions (Veteli *et al.* 2002; Najar *et al.* 2014) and seasonality (Osier *et al.* 2000). Whether root and shoot defensive chemistry changes in concert or independently in response to these basic ecological factors is unclear (Wurst *et al.* 2008).

The defensive chemistry of members of the genus *Populus* has been very well studied, in particular trembling aspen, *Populus tremuloides*, providing a robust foundation upon which to test ideas related to leaf-root defence integration (Lindroth and St. Clair 2013). Trembling aspen produces two major groups of secondary chemicals, phenolic glycosides and condensed tannins (Lindroth and Hwang 1995; Barbehenn and Peter Constabel 2011), concentrations of both can be quite high, though neither is consistently more abundant than the other. Condensed tannins are class of carbon-based anti-nutritive defensive chemicals that despite their high concentrations and up-regulation following defoliation events are not considered to significantly affect the performance of lepidopteran herbivores in aspen (Lindroth and St. Clair 2013), though they have been indicated to reduce food quality (Lindroth *et al.* 1999). Condensed tannins have also been documented to negatively impact fungal endophytes in *Populus* hybrids (Bailey *et al.* 2005) and reduce protein digestion in mammalian herbivores in various plant species (Barbehenn and Peter Constabel 2011). Condensed tannins may also play a role in nutrient cycling in aspen forests, although there have been few empirical tests (Schweitzer *et al.* 2008; Madritch and Lindroth 2015).

Despite our mixed understanding of their ecological significance, aspen allocate a substantial portion of their resources to the production of condensed tannins in leaves; up to 20 % of dry leaf weight (Lindroth and Hwang 1995). Aspen leaf chemistry is also highly plastic, affected by many factors including plant age (Donaldson *et al.* 2006b), leaf age (Osier *et al.* 2000), drought (Greitner *et al.* 1994; Roth *et al.* 1997; Kanaga *et al.* 2009), herbivory (Lindroth and Hwang 1995; Major and Constabel 2007; Lindroth and St. Clair 2013), shading (Lindroth and Hwang 1995; Hemming and Lindroth 1999; McDonald *et al.* 1999; Agrell *et al.* 2000; Osier and Lindroth 2006), competition (Donaldson *et al.* 2006a) and nutrient availability (Donaldson *et al.* 2006a; Osier and Lindroth 2006; Najar *et al.* 2014). In contrast, aspen root chemistry is less well understood; only two studies have been conducted, both on young aspen, showing that condensed tannin levels occur at levels around 4–5 % of dry root weight, and decrease in response to increased nutrient availability (King *et al.* 2005; Stevens *et al.* 2014). Studies investigating condensed tannin concentrations in aspen roots in either naturally occurring or mature trees are as far as we can tell, absent from the literature.

In this study, we use leaf and fine root tissue from mature, naturally occurring aspen to answer the following three questions: (i) Within a single aspen stem, are root tannin levels correlated with leaf tannin levels? (ii) How do root and leaf tannins vary with time, stand position (edge vs. interior), and stem diameter? (iii) Is the ratio of root to leaf tannins within a single stem consistent over time?

## Methods

### Location and sampling design

This study took place at the Roy Berg Kinsella Research Ranch in central Alberta, Canada (53.08532 N, 111.5636 W). The ranch is located in the aspen parkland ecoregion, and is made up of a mixture stands of trembling aspen and rough fescue grassland (Lamb *et al.* 2009). The aspen parkland is at the edge of suitability for aspen, and these stands are small and fairly uniform, with similar levels of light penetration at the edge and interior of a stand.

In June 2015, we identified three stands of trembling aspen a minimum of 35 m apart with no evidence of aspen suckering in the grassland areas between the stands. We sampled from several stands as aspen is a clonal species that propagates through root suckering, and many of the individual aspen ‘trees’ in a stand could be clonal ramets of the same genet, which can cover large areas and live for thousands of years (Mitton and Grant 1996). This study focuses on the relationship between leaf and root tannins in naturally occurring aspen, so the frequent approach of using an artificially created common garden with propagated clones would not be feasible, despite the advantage of controlling for genetic relatedness between individuals. Using naturally occurring aspen, despite our best efforts to sample aspen in different stands, and different parts of stands, we do not know how the aspen we sampled are related. Therefore, we will use the term stem to refer to our sample individuals throughout this paper, as opposed to tree (which suggests no genetic relationship) or ramet (which suggests a clonal relationship). Within each of our identified stands, we selected six aspen stems, stratified into three pairs, spread around the perimeter of the stand. These pairs consisted of one stem from the edge of the stand and one from the interior. This allowed us to examine the effects of stand position, which may have differing light levels, on tree chemistry. We defined edge as stems within 1 m of the adjacent grassland, with the interior as a minimum of 6 m from the edge stem towards the centre of the stand. All 18 stems were between 3.5 and 9 cm diameter at breast height.

To compare condensed tannin levels in different tissue types, we collected both leaf and fine root tissue from each individual stem. For the leaves, 5–10 mature leaves with no visible evidence of disease or damage were collected from the mid-crown of the stem using a pole pruner. For roots, ~5 g of fine roots were collected by digging around the base of the focal stem until we found a lateral root, and then following this until we located fine root tissue to ensure the collected root tissue originated from the focal stem.

To examine the effect of season on tannin concentrations, leaf and root tissue from the 18 focal stems were re-sampled on 13 August and 13 October in the same manner as described above. Including the initial June sampling, these three sample periods were selected to align with the early growing season (post leaf flush), mid-growing season and leaf senescence, respectively. To control for induction of secondary chemicals due to previous samplings, a new neighbouring stem, nearest in proximity and size to each focal stem, was also selected at each subsequent sampling as a control tree, bringing the total number of stems sampled to 54.

Across all sampling, stems were visually surveyed for health and stems with visible foliar or trunk damage were excluded from the study.

### Chemical analysis

Samples were placed on ice and transported to −80 °C storage the same day as harvest. They were lyophilized for 48 h, and ground using 2 mm beads in a Qiagen TissueLyser II (Qiagen, Hilden, Germany) with bead mill at 30 rpm. The HCl-butanol method (Hagerman and Butler 1980; Porter *et al.* 1985) was used to analyse both leaf and root samples for condensed tannins. Ground tissue was weighed to within 5 % of 30 mg and the exact weight was recorded. This ground tissue was then extracted twice using 70 % acetone with ascorbic acid. For aspen leaf tissue, initial tests showed that the concentration of condensed tannins in the supernatant was above the detection limit of the spectrophotometer, so the leaf tissue supernatant was diluted to 20 %. Sample extracts were then reacted with the butanol-HCl reagent (5 % 12 M HCl in 95 % butanol) as well as a solution of 2 g ferric ammonium sulfate dissolved in 100 mL of 2 M HCl as recommended by Porter *et al.* (1985). The ratio of reagents was 5:30:1 (sample supernatant:butanol-HCl:ferric solution). This sample was left to react for 50 min at 95 °C, then absorbance at 550 nm was measured using a BioTek PowerWave XS Spectrophotometer (BioTek Instruments, Inc., Winooski, VT, USA). Condensed tannins were purified from leaves of natural trembling aspen collected on the University of Alberta campus (~150 km from the study site) following the method of Hagerman and Butler (1980) and used as standards. The spectrophotometer readings were converted into milligrams of tannins per gram of tissue using a standard curve, with each sample value adjusted for initial sample weight and any dilution.

### Statistical analysis

To determine if previous sampling affected current chemistry, we compared the focal stems at each time point to the paired sampling control stems using an ANOVA. To determine if leaf and root condensed tannin levels were correlated, and if time, stand position and stem size influenced root chemistry, a linear mixed model was run with root condensed tannin concentrations as the response variable, while fixed factors were leaf tannin concentrations, season (as a repeated measure), diameter and stand position (edge or interior). Edge and interior pairs, nested within stand, served as a random effect. To determine if time, stand position and stem diameter influenced leaf chemistry, a linear mixed model was run with leaf condensed tannins as the response variable, while fixed factors were season (as a repeated measure), size and stand position, with edge and interior pairs nested within stand as a random effect. To determine if leaf and root tannins co-varied over time, the ratio of root:leaf (R/L) tannins was calculated and analysed using a mixed effect model, with season (repeated measure), position in stand and size as fixed effects and pairs nested within stands as a random effect. Statistics were done in SPSS v. 25 (IBM Corp. 2016), graphs were created in Sigma Plot v. 11 (Systat Software Inc. 2008).

## Results

The sampling control stems were not significantly different from paired focal stems (df = 1, *F* = 0.062, *P* = 0.980), which indicated that intervals between our repeated sampling points were long enough to avoid capturing any effects of induction due to previous sampling in our later time points.

**Figure 1.**
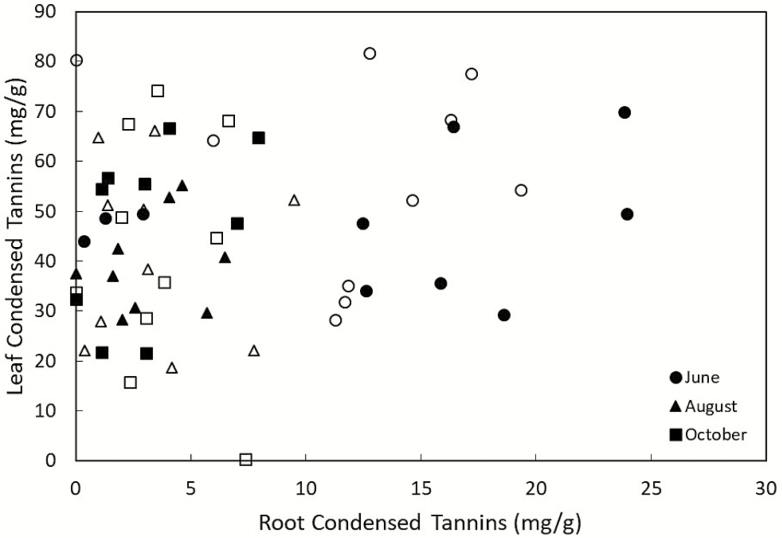
Root versus leaf condensed tannins (mg g^−1^ tissue) for individual *Populus tremuloides* stems. Symbol shape denotes the time period during which the tissue was sampled, closed circles (●)indicate stems in the interior of the stand and open circles (○) indicate stems in the edge of the stand.

**Figure 2.**
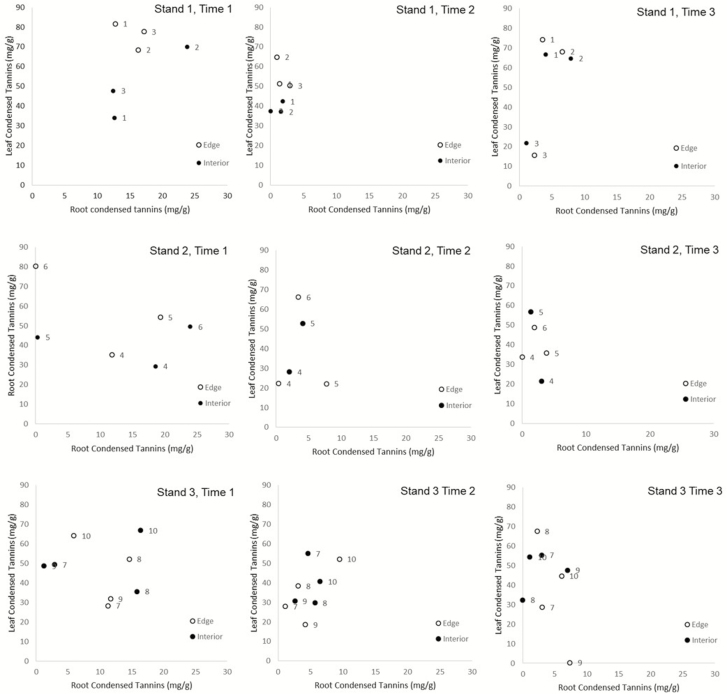
Fine root condensed tannins versus leaf condensed tannins for each stand and time period, with edge and interior trees separated by colour. The numbers next to each point indicate matching pairs of edge and interior trees.

**Figure 3.**
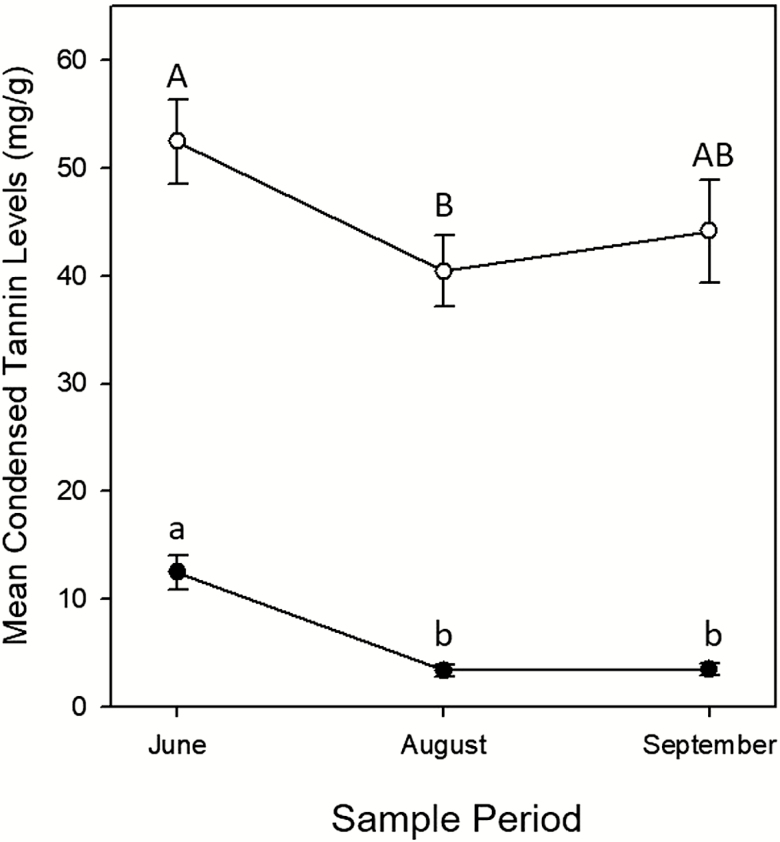
Average condensed tannin levels (mg g^−1^) for roots (●) and leaves (○) across three time points in *Populus tremuloides* (means ± SE). Capital letters indicate significant differences (*P* < 0.05) between leaf values, and lowercase letters indicate differences between root values.

Root tannin levels were not correlated with leaf tannin levels within a stem (Table 1; Fig. 1). Stem diameter and stand position did not affect root or leaf tannin concentrations (Table 1; Fig. 2 for root and leaf tannin levels graphed by time, stand and stand position). Time altered both root and leaf condensed tannin concentrations, with the highest concentrations of both occurring in June, dropping in August and then rising slightly in October for leaves though tannin concentrations in root stayed consistent from August to October (Table 1; Fig. 3).

**Table 1.** Leaf and root condensed tannin levels as a function of time, stand position and tree diameter. Generalized linear mixed model models were used to test for the effects of listed factors on leaf and root condensed tannins.

Factor	Root condensed tannins			Leaf condensed tannins		
	df	*F*	*P*	df	*F*	*P*
Leaf condensed tannins	1	0.111	0.741	–	–	–
Time	2	12.726	0.0001	2	4.59	0.016
Stand position	1	0.328	0.569	1	0.280	0.599
Tree diameter	1	0.126	0.724	1	0.133	0.717

**Figure 4.**
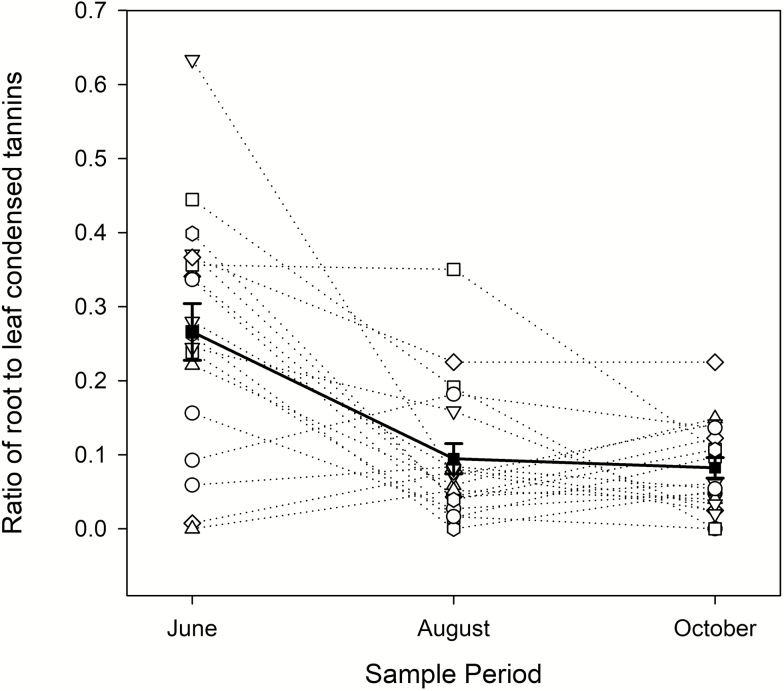
Ratio of fine root condensed tannins (mg g^−1^ tissue) to leaf condensed tannins (mg g^−1^ tissue) in *Populus tremuloides* collected over three time points. The unfilled points represent individual stems, with samples from the same stem connected with dashed lines. The filled markers (■) and solid line represent the mean ratio for each time point, with error bars indicating ±SE.

The ratio of root to leaf tannins in a single stem was not consistent over time, with more allocations to root relative to leaf in June as compared to August or October (Table 2; Fig. 4).

**Table 2.** Ratio of root:leaf condensed tannins as a function of time, stand position and tree diameter. A generalized linear mixed model was used to test for the effects of the listed factors on root:leaf ratio.

Source	Root:leaf ratio of condensed tannins		
	df	*F*	*P*
Time	2	6.904	0.002
Stand position	1	0.111	0.597
Tree diameter	1	0.481	0.972

## Discussion

We found that absolute concentrations of leaf and root tannins were different within our sampled aspen stems (roots contained ~85 % less tannins than leaves), which is consistent with existing literature (Kosola *et al.* 2004, 2006; King *et al.* 2005; Stevens *et al.* 2014). We also found that relative concentrations of condensed tannins in roots were not correlated with leaf concentrations from the same stem, and that the ratio of root to leaf tannins within a stem was not consistent over time. Root tannin concentrations were highest relative to leaves in June, and lower in August and October.

These results address a long-standing hypothesis in ecology that root and leaf defensive traits should be similar as both tissue types experience similar levels of herbivory and are similarly crucial the survival of a plant (Kaplan *et al.* 2008; Kembel and Cahill 2011; Parker *et al.* 2012). The observation that in our sampled stems, condensed tannin concentrations are not linked between leaves and fine roots, and that relative concentrations of condensed tannins in leaves and roots may change over time disagrees with the hypothesis outlined above. Parker *et al.* (2012) suggested that comparing patterns of plant chemistry in above- and below-ground tissues can also indicate the strength of forces that drive natural selection of plant chemical traits. Our results indicate that the evolutionary pressures experienced below-ground may be weaker, as they have not selected for the same the high concentrations of condensed tannins found above-ground.

The predictions made by Kaplan *et al.* (2008) are based on the optimal defence theory (McKey 1979; Rhoades 1979); however, despite our results this theory might still be applicable below-ground if we consider that below-ground tissues may not be vulnerable in the same way as above-ground tissues, or that the defences we measure in above-ground tissues may not function the same way in deterring below-ground herbivores. Therefore, we suggest additional research to determine if below-ground tissues are less vulnerable to herbivory, or to show that defence chemicals work differently to deter below-ground herbivores.

We also found that there was as much variation in condensed tannin production within a stand, which is possibly one large clonal organism, as there was between stands. If a stand represents one large clonal organism, then this result contrasts previous data that shows that variation between clones is higher than variation within clones (Donaldson and Lindroth 2007); however, it is also possible that the variation we see within stands suggests that the stands are not in fact single clones, but several individuals together, which would put this finding more in line with existing research.

Our finding that, across sampled stems, leaf condensed tannins are highest in the early summer with lower levels later in the season agrees with the published results on aspen leaf chemistry (Lindroth and Hwang 1995; Roth *et al.* 1998; Osier *et al.* 2000). Stem diameter and stand position were not significantly related to leaf tannins of aspen sampled in this study. Osier *et al.* (2000), also following the rationality of optimal defence theory, suggested that a slight decrease in food quality that comes with elevated aspen condensed tannin concentrations could account for higher observed levels of condensed tannins during June, when population densities for aspen herbivores are at their peak (Parry *et al.* 1998). Our observed pattern could also be driven by changes in resource availability from stored resources which can influence poplar defensive chemistry (Najar *et al.* 2014) or changes in seasonal allocation patterns between growth and defence. The slight increase in leaf tannins in October also agrees with work suggesting that autumn cold was related to an increase in the concentration of tannins in poplars (Li *et al.* 2011), as well as observations that intense frost-defoliation can cause increased levels of condensed tannins in aspen foliage (St. Clair *et al.* 2009), which can be explained by studies indicating that phenolic compounds can aid in cold tolerance (Koyama *et al.* 2014).

While previous studies have indicated that aspen leaf tannins can vary over a growing season, to our knowledge, none have examined how root tannin levels vary across the same time period. Our observed pattern that across all stems, fine root condensed tannin concentrations changed over the growing season, with highest levels in June and lower levels in August and October suggests that there might be some population level factor that is the driver of this pattern. Of the major below-ground herbivores of aspen present in Alberta, ghost moth (*Sthenopis purpurascens*, Lepidoptera: Hepialidae), aspen root girdler (*Agrilus horni*, Coleoptera: Buprestidae) and poplar-butt borer (*Xylotrechus obliteratus*, Coleoptera: Cerambycidae), only poplar-butt borer exhibits feeding below-ground during the early part of the growing season (Nord *et al.* 1965; Flaim and Platt 1999; Steed and Burton 2015); however, these herbivores are severely understudied. Of the three, only ghost moth was observed at our site during this experiment, though not on any focal stems. There are also root pathogens of aspen, such as *Armillaria* sp.; however, these were not observed at the study site. Similar to above-ground tissues, resource availability could also play a role in the observed below-ground seasonal pattern (Najar *et al.* 2014).

## Conclusions

Our observation that root condensed tannin concentrations were not related to leaf condensed tannins within individual aspen trees indicates that aspen leaf and root defensive traits may not be strongly related in individual aspen. This suggests separate evolutionary drivers above- and below-ground. However, similar patterns in roots and shoots over time, with highest levels in June and lower levels in August and October suggest that at the population level, there may be similar environmental drivers for population-level patterns. Overall, more studies of leaf and root defensive traits are needed to clarify the extent to which selection acts at the whole-plant level for these traits, or independently for different tissues. Our general finding that fine root condensed tannins were different from leaf condensed tannins suggests that the theories currently in use for above-ground plant defence may need some tweaking before they can be widely used to predict below-ground traits.

## Sources of Funding

This work was funded by NSERC Discovery and NSERC Discovery Accelerator grants to J.F.C., a University of Alberta Undergraduate Research Initiative grant to V.M. and a Queen Elizabeth II Scholarship to M.A.D.

## Contributions by the Authors

M.A.D., V.M., N.E. and J.F.C. contributed to the design of this experiment. Field work and chemical analysis were done by M.A.D. and V.M. M.A.D., V.M., N.E. and J.F.C. contributed to writing.

## Conflict of Interest

None declared.
